# Aggressive pituitary tumours and carcinomas, characteristics and management of 171 patients

**DOI:** 10.1530/EJE-22-0440

**Published:** 2022-08-26

**Authors:** Pia Burman, Jacqueline Trouillas, Marco Losa, Ann McCormack, Stephan Petersenn, Vera Popovic, Marily Theodoropoulou, Gerald Raverot, Olaf M Dekkers, the ESE survey collaborators

**Affiliations:** 1 Department of Endocrinology, Skåne University Hospital Malmö, University of Lund, Lund, Sweden; 2 Faculty of Medicine Lyon-Est, University Claude Bernard Lyon 1, Lyon, France; 3 Marco Losa Department of Neurosurgery, IRCCS San Raffaele Scientific Institute, Vita-Salute San Raffaele University, Milan, Italy; 4 St Vincent’s Hospital and Garvan Institute of Medical Research, Sydney, Australia; 5 ENDOC Center for Endocrine Tumors, Hamburg, Germany; 6 University Belgrade, Belgrade, Serbia; 7 Medizinische Klinik und Poliklinik IV, LMU Klinikum, Ludwig-Maximilians-Universität München, Germany; 8 Fédération d’Endocrinologie, Groupement Hospitalier Est, Hospices Civils de Lyon, University of Lyon-Est de Lyon, Bron, France; 9 Department of Internal Medicine (Section Endocrinology) & Clinical Epidemiology, Leiden University Medical Centre, Leiden, The Netherlands

## Abstract

**Objective:**

To describe clinical and pathological characteristics and treatment outcomes in a large cohort of aggressive pituitary tumours (APT)/pituitary carcinomas (PC).

**Design:**

Electronic survey August 2020–May 2021.

**Results:**

96% of 171 (121 APT, 50 PC), initially presented as macro/giant tumours, 6 were microadenomas (5 corticotroph). Ninety-seven tumours, initially considered clinically benign, demonstrated aggressive behaviour after 5.5 years (IQR: 2.8–12). Of the patients, 63% were men. Adrenocorticotrophic hormone (ACTH)-secreting tumours constituted 30% of the APT/PC, and the gonadotroph subtypes were under-represented. Five out of 13 silent corticotroph tumours and 2/6 silent somatotroph tumours became secreting. Metastases were observed after median 6.3 years (IQR 3.7–12.1) from diagnosis. At the first surgery, the Ki67 index was ≥3% in 74/93 (80%) and ≥10% in 38/93 (41%) tumours. An absolute increase of Ki67 ≥ 10% after median of 6 years from the first surgery occurred in 18/49 examined tumours. Tumours with an aggressive course from outset had higher Ki67, mitotic counts, and p53. Temozolomide treatment in 156/171 patients resulted in complete response in 9.6%, partial response in 30.1%, stable disease in 28.1%, and progressive disease in 32.2% of the patients. Treatment with bevacizumab, immune checkpoint inhibitors, and peptide receptor radionuclide therapy resulted in partial regression in 1/10, 1/6, and 3/11, respectively. Median survival in APT and PC was 17.2 and 11.3 years, respectively. Tumours with Ki67 ≥ 10% and ACTH-secretion were associated with worse prognosis.

**Conclusion:**

APT/PCs exhibit a wide and challenging spectrum of behaviour. Temozolomide is the first-line chemotherapy, and other oncological therapies are emerging. Treatment response continues to be difficult to predict with currently studied biomarkers.

## Introduction

A small proportion of pituitary tumours are aggressive and progress despite standard treatment*.* About 1–2 in 10 will metastasize and then be classified as carcinomas (1). To gain a broader knowledge of the treatment of these underappreciated and challenging tumours, a European Society of Endocrinology (ESE) task force conducted a survey in 2015–2016 (2) and subsequently developed a guideline recommending temozolomide (TMZ) as first-line chemotherapy (3). Several issues remained unsolved, such as the effect of combination with radiotherapy (RT) and/or other drugs, the effects of emerging treatments, the value of RT, and, in carcinomas, the effect of loco-regional treatment of metastases.

The aims of the present survey were to address unresolved issues, to describe in detail the clinical and pathological characteristics of the tumours at diagnosis, the evolution over time, the pattern of metastatic behaviour , survival, and update/extend the outcome of treatments.

## Methods

A second patient survey was developed by the ESE taskforce on Aggressive Pituitary Tumours/Carcinomas and made available from August 2020 to the participants of the ESE 2015–2016 survey. In addition to providing more information on patients included in the first ESE survey, participating centres could also report new patients, provided that they applied the ESE guidelines (3) definition of APT: invasive tumour with an unusually rapid growth rate, or a tumour not controlled by repeat surgery, RT, and/or demonstrating resistance to medical treatments. The survey was conducted via an online form and stored in password-protected files. The methods and procedures for collecting and collating patient data adhered to EU GDPR (European Union General Data Protection Regulation) for Special category data (Articles 6.1 and 9). Completed forms were sent to the ESE office and the database was closed on 1 May 2021.

Clinical and pathological data on tumours, treatments, and survival were collected. Volumetric assessment of the lesions was performed, and modified Response Evaluation Criteria In Solid Tumours (RECIST) criteria were applied for effect evaluation. Complete response (CR) was defined as no visible tumour, partial response (PR) as at least 30% tumour regression, stable disease (SD) as less than 30% regression but no more than 10% increase, and progressive disease (PD) as more than 10% increase in tumour size (or new metastatic deposits).

### Statistical analysis

Data were described using proportions and means or medians depending on the underlying distribution. For time-to-event analysis, the Kaplan–Meier method was used.

## Results

### Patient cohort

#### Clinical and radiological presentation at diagnosis

A total of 171 patients (107 men), 121 aggressive pituitary tumours (APT) and 50 pituitary carcinomas (PC), were included (Table 1) from 15 European countries, Argentina, Australia, Brazil, Columbia, Japan, Israel, and the United States. From these, 87 (63 APT, 24 PC) participated in the ESE 2015–2016 survey (cohort 1) of which 58 (13 PC) were alive at the end of the study and follow-up data over an additional mean of 3.3 years were reported to the present study, while 83 (57 APT, 26 PC) were new (cohort 2). Since the two cohorts (Supplementary Table, see section on supplementary materials given at the end of this article) had comparable clinical characteristics, only combined data are presented.

**Table 1:** Characteristics at diagnosis of 171 aggressive pituitary tumours (APT) and pituitary carcinomas (PC) included in ESE survey 2020–2021. Data are presented as *n* (%) or as mean ± s.d.

Characteristics	APT	PC	*P* value
*n*	121	50	
Age at diagnosis	44.6 ± 16.3	48.4 ± 14.0	0.14
Patients’ sex			0.80
Female	44 (36.4%)	19 (38.0%)	
Male	76 (62.8%)	31 (62.0%)	
Missing	1 (0.8%)	0 (0.0%)	
Initial hormone secretion^a^			0.57
None	33 (27.3%)	12 (24.0%)	
Prolactin	38 (31.4%)	16 (32.0%)	
ACTH	32 (26.4%)	19 (38.0%)	
FSH	1 (0.8%)	0 (0.0%)	
GH	12 (9.9%)	3 (6.0%)	
TSH	3 (2.5%)	0 (0.0%)	
Unknown	2 (1.7%)	0 (0.0%)	
MRI at initial diagnosis			0.19
Microadenoma	5 (4.1%)	1 (2.0%)	
Macroadenoma	82 (67.8%)	42 (84.0%)	
Giant adenoma (≥40 mm)	31 (25.6%)	6 (12.0%)	
Missing	3 (2.5%)	1 (2.0%)	
Part of hereditary syndrome?			0.97
No	116 (95.9%)	48 (96.0%)	
Yes	2 (1.7%)	1 (2.0%)	
Unknown	3 (2.5%)	1 (2.0%)	
Invasive at diagnosis Yes/No			0.96
Not invasive^b^	19 (15.7%)	7 (14.0%)	
Invasive	76 (62.8%)	32 (64.0%)	
Unknown	26 (21.5%)	11 (22.0%)	
Was pituitary radiotherapy performed?			0.051
No	17 (14.0%)	1 (2.0%)	
Yes	103 (85.1%)	49 (98.0%)	
Unknown	1 (0.8%)	0 (0.0%)	
Was pituitary surgery performed?			0.40
No	9 (7.4%)	2 (4.0%)	
Yes	112 (92.6%)	48 (96.0%)	

^a^Co-secretion of hormones in eight cases, see Results‘ section; ^b^invasion refers to into the cavernous sinus or bone.

**Figure 1:**
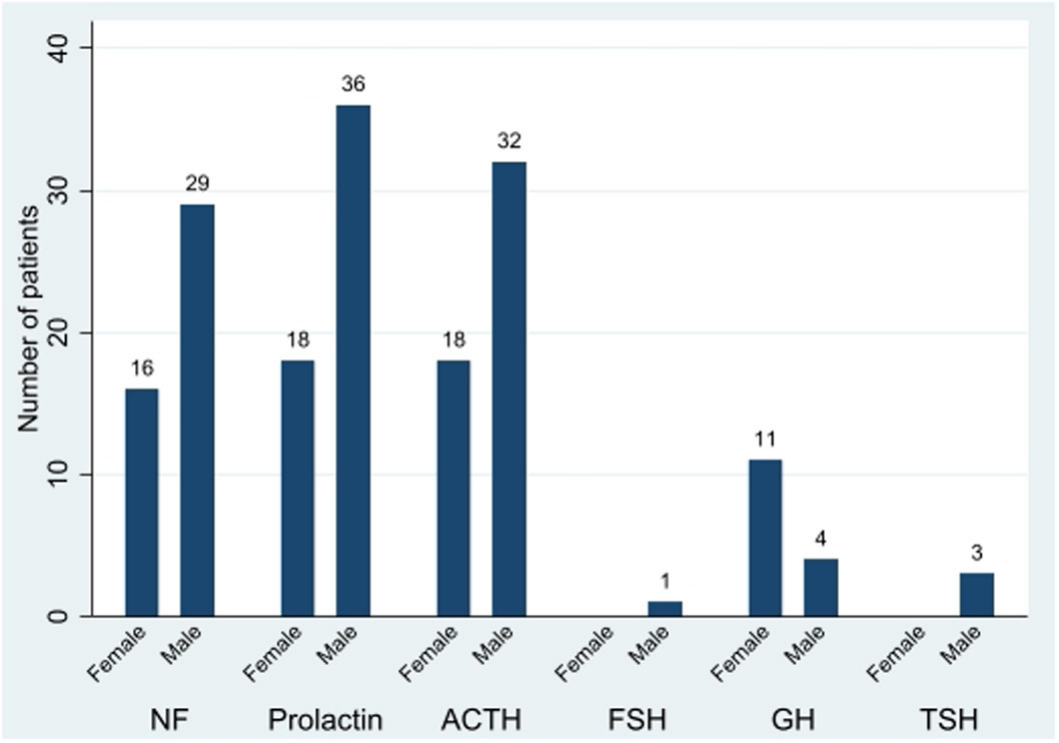
Hormone secretion and gender reported in 168 aggressive pituitary tumours/carcinomas. Male gender was predominant among tumours secreting PRL and ACTH, and in non-functioning tumours (e.g. clinically silent tumours regardless of hormone staining at immunohistochemistry); number of cases in columns.

Mean age at diagnosis was 46 years (range 3–74 years); 32% of the tumours were prolactin (PRL)-secreting, 30% adrenocorticotrophic hormone (ACTH)-secreting, and 27% clinically non-functioning (NF) (Fig. 1). Co-secretion of hormones was observed at diagnosis in eight patients, PRL/GH (*n* = 4), thyroid-stimulating hormone (TSH)/growth hormone (GH) (1), TSH/follicle-stimulating hormone (FSH), (TSH-driven hyperthyroidism and enlarging testes; lineage transcription factor steroidogenic factor 1 (SF-1) was positive, pituitary-specific positive transcription factor 1 (Pit-1) and TSH immunohistochemistry were not investigated) (1), FSH/luteinizing hormone (LH) (*n* = 1), and PRL/glycoprotein α-subunit (8.8, ref <0.7 IU/L) (*n* = 1).

At initial presentation, 97 tumours (29 PC) were considered clinically benign while 65 (20 PC) demonstrated aggressive behaviour from the outset (unknown in 9). An aggressive phenotype from outset was observed in 28% of ACTH-secreting, 32% of NF, 46% of prolactinomas, 86% of the GH-secreting, and in 2/3 TSH-secreting tumours.

**Figure 2:**
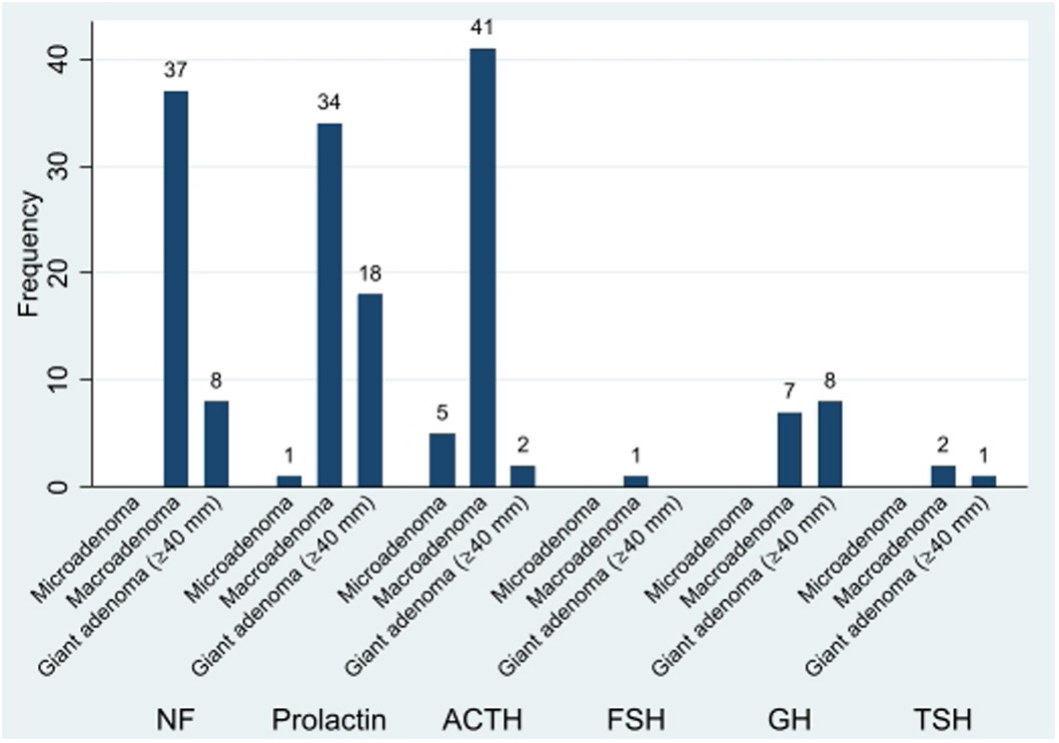
Tumour size at initial presentation with respect to the main hormone secreted in 165 patients with APT/PC (information in hormone secretion or tumour size missing in 6).

Most APT/PC presented as macroadenomas (*n* = 125/168) and giant tumours (*n* = 37), and 6 were microadenomas (5 ACTH-secreting) (Fig. 2). One hundred eight out of 134 were invasive into the cavernous sinus and/or bone, while 26/134, including 4/5 ACTH-secreting microadenomas, were not.

### Proliferative markers and immunohistochemistry

**Figure 3:**
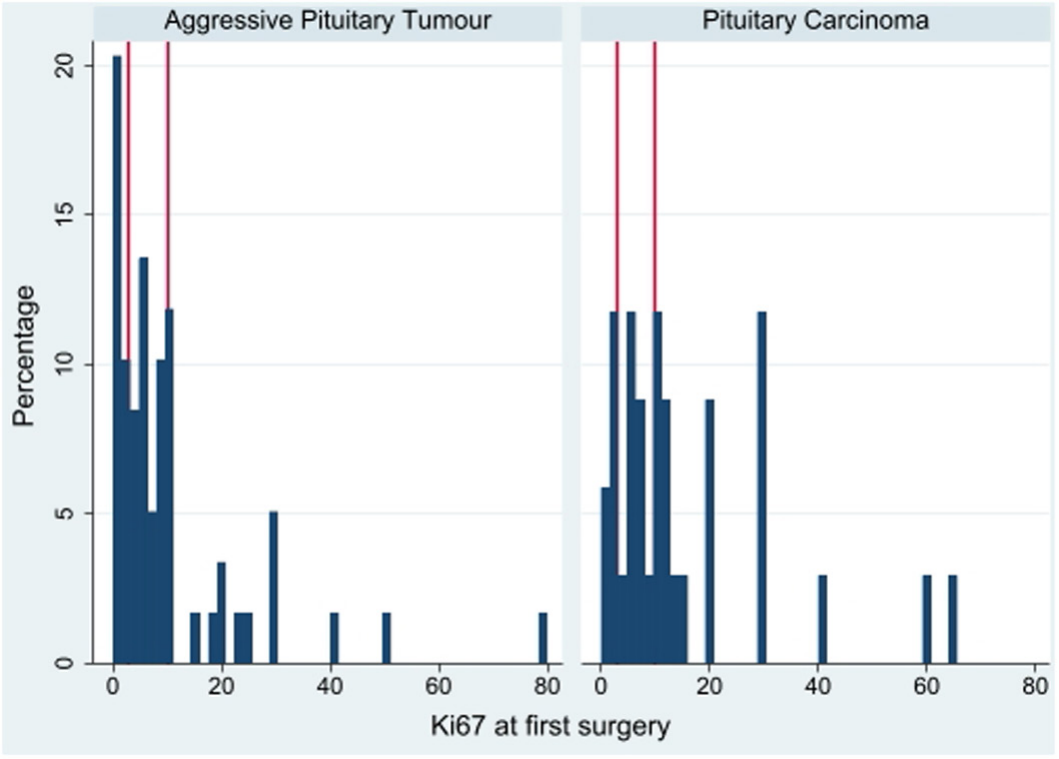
Histogram showing the distribution of Ki67 values at first surgery for aggressive pituitary tumours (left panel) and pituitary carcinomas (right panel).

Median Ki67 index determined at the first surgery was 6% (IQR: 3–10) in APT and 10% (IQR: 5–20) in PC (Fig. 3 and Table 2). The proportions of tumours with Ki67 ≥ 3% were not substantially different across tumour subtypes. The proportion with Ki67 ≥ 10% was 29% in NF tumours and 44% in functioning tumours (*P* = ns), and higher in PC (56%) compared to APT (32%, *P* = 0.03).

**Table 2:** Summary of available observations on markers of proliferation; Ki67 index, mitotic count, and p53 expression at the first surgery in aggressive pituitary tumours (APT) and pituitary carcinomas (PC).

	APT	PC
Ki67 index (%)		
Tumours tested	58/112	35/48
<3, *n* (%)	13 (22.4)	6 (17)
≥3, *n* (%)	45 (77.5)	29 (82.3)
≥10, *n* (%)	19 (32.7)	19 (54.2)
Mitotic count		
Tumours tested	26/112	18/48
*n* ≤ 2, *n* (%)	18 (69)	8 (44)
*n* > 2, *n* (%)	8 (31)	10 (55)
p53 expression (%)		
Tumours tested	24/112	19/48
<10, *n* (%)	18 (75)	12 (63.2)
≥10, *n* (%)	6 (25)	7 (36.8)
≥20, *n* (%)	3 (12.5)	5 (26.3)
≥30, *n* (%)	0	4 (21.1)
≥40, *n* (%)	0	3 (15.8)

Four tumours (3 PC) with p53 expression were not included in this table. The positive staining was reported to be weak in two, strong in one and only as being ‘positive’ in one, the percentage of positive nuclei was not given.

Mitotic counts did not differ between APT and PC. p53 immunostaining tended to be higher in PC compared to APT (Table 2). p53 ≥ 10% was associated with higher Ki67 indices compared to p53< 10% (50th percentile 30% vs 9%). Proliferative markers were generally lower in PCs with an initial clinically benign behaviour compared to PC with an aggressive course from outset (Table 3).

**Table 3:** Pituitary carcinomas with clinically aggressive and clinically benign behaviour from outset according to the opinion of the clinicians.

	Clinical behaviour	
	Aggressive	Benign
Total, *n*	20	29
Males	14	16
Females	6	13
NF, %	15	31
Time to metastases	4.1 (2.4–6.0)	8.5 (5.9–14.8)
Ki67 index (%)	<3 (1/18)	<3 (5/17)
Median (IQR)	14 (7–30)	6.5 (2–11)
Mitotic count, (*n*/10 HPFs)	≤2 (2/8)	≤2 (8/10)
Median (IQR)	5 (3–8.5)	0 (0–2)
P53 staining (%)	≤10 (4/9)	≤10 (5/7)
Median (IQR)	15 (7–35)	3 (0–10)

HPFs, higher power magnification fields; NF, non-functioning pituitary tumours.

Immunostaining of hormones was performed in 39 of 42 operated NF. Twenty-five demonstrated staining results as follows: ACTH only (9), GH only (6), TSH only (1), FSH only (1), and Pit-1 (3). Some exceptional plurihormonal tumours from different lineages were also observed; two ACTH/LH ± α-subunit glycoprotein, two ACTH/GH ± PRL, one TSH/FSH/LH (SF-1, but not Pit-1 positive). Fourteen NF tumours were immunonegative for all hormones tested, and 4/14 had additional transcription factor staining and tested negative for SF-1, Pit-1, and TPIT, thus classified as null-cell tumours.

### Clinical behaviour of the tumours and Ki67 proliferative indices at follow-up

The 97 tumours initially considered to be clinically benign displayed an aggressive course after a median of 5.5 years (IQR 2.8–12 years), maximum 33 years. The time interval was similar for APT and PC. Four of the five ACTH-secreting microadenomas had evolved into macroadenomas, a PRL-secreting microadenoma into a dopamine-resistant PC.

Seven NF tumours became clinically functioning; 5/13 silent corticotroph and 2/6 silent somatotroph tumours, at a median of 11 (range 3–14) years after diagnosis (Table 4). The patients had multiple surgeries, and proliferative tumour markers increased over time. One initially TSH/GH- secreting APT became clinically silent after 8 years.

**Table 4:** Characteristics of seven clinically silent pituitary tumours that became clinically functioning.

Gender	Age at diagnosis, years	APT/PC	Type of silent tumour	op/RT, *n*	Hormone staining at IHC	Time to hormone secretion (years)	Hormone secreted (× ULN)	p53 (%)	Ki67 index (%)	Mitotic count (*n*/10 HPF)
Male	21	APT	Corticotroph	5/2	ACTH, PRL, GH	14	ACTH (×1)	nd	nd	nd
Male	41	APT	Corticotroph	3/0	ACTH	3	ACTH (×3)	nd	10--18	5--5
Female	28	PC	Corticotroph	5/1	ACTH, LH, α-SU	13	ACTH value missing	nd--*10	5	nd--*3
Female	38	PC	Corticotroph	3/3	ACTH	3	ACTH (×3.5)	8--5/10 HPF	7--25	nd--1
Male	46	PC	Corticotroph	3/1	ACTH	6	ACTH (×10)	60	5--*25	nd
Female	58	PC	Somatotroph	3/1	nd--*Pit1 pos	13	GH: 7 µg/L; IGF-1 (×3.5)	nd--pos.	nd--9	nd--3
Female	40	PC	Somatotroph	4/1	GH	11	GH?; IGF-1: 109–278 µg/L (<255)	neg.--*10	4--10	nd

α-SU, α-subunit glycoprotein; HPF, high power fields; IHC, immunohistochemistry; nd, not done/unknown; Op, operations; RT, radiotherapy; ULN, upper limit of normal; --, findings at 1st to last op. before the change from silent to functioning tumour; *at a surgery performed after the change from silent to functioning tumour.

In patients who had repeat surgery, Ki67 increased over time with at least 10% in 18/49 examined tumours after a median of 6 (range: 1–11) years from the first surgery. The last Ki67 in APT was median 10% (IQR: 4–17.5) and 20% in PC (IQR: 10–27.5).

Bilateral adrenalectomy (BADX) was performed in 22 (16 APT, 6 PC) of 50 patients with Cushing’s disease. Five clinicians considered that an initially clinically benign tumour demonstrated aggressive growth only after BADX, four clinicians considered that an aggressive growth from diagnosis was accelerated by BADX, five clinicians considered that the course was aggressive already from the diagnosis, and eight clinicians considered that an effect of BADX could not be determined. Compared to 6 months prior to BADX, an increase of circulating ACTH, considered to be compatible with tumour progression (4) during the following 3–6 months, was seen in 2/8 APT and in 2/5 PC. In another two PC with marked ACTH elevations after BADX (475 × ULN and 2195 × ULN), metastases had been observed around the time of adrenal surgery.

### Metastases

**Figure 4:**
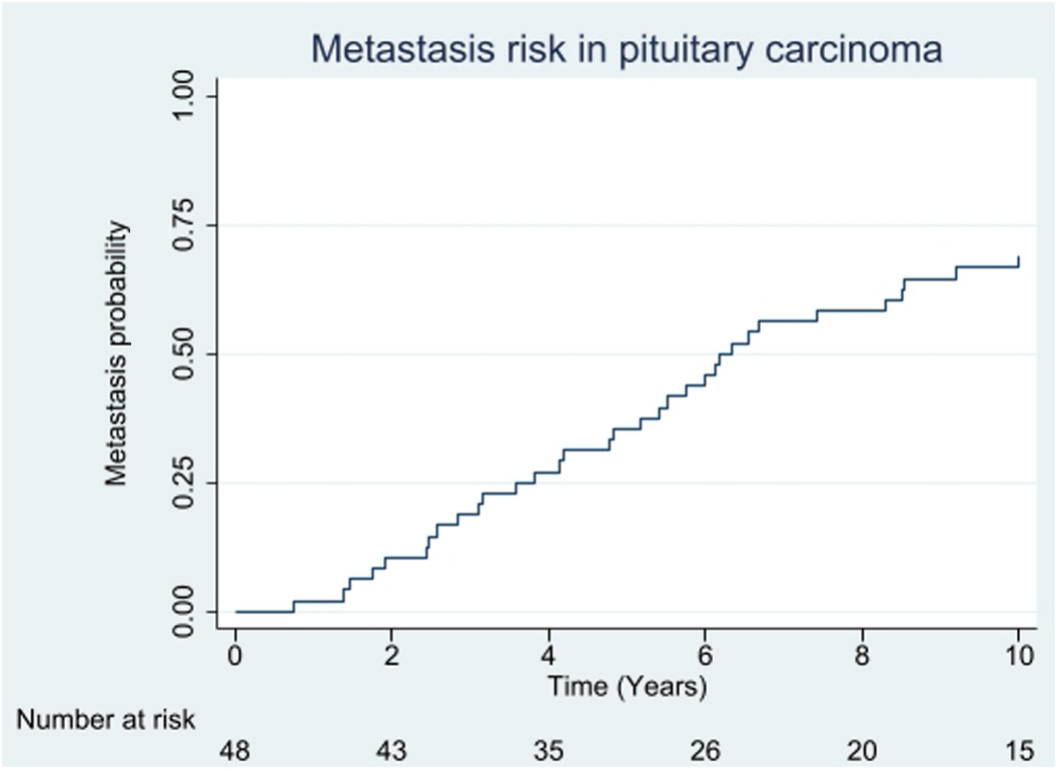
Kaplan–Meier curve showing time from diagnosis of the pituitary tumour to detection of metastasis in 48 patients with pituitary carcinoma.

Metastatic spread was observed after a median of 6.3 (IQR: 3.7–12.1), maximum 36 years after diagnosis of the pituitary tumour (Fig. 4), and 3.8 (IQR: 1.5–5.9 years) years from the development of clinically aggressive behaviour. The time tended to be shorter in PC with Ki67 ≥ 10% vs <10%, median 3.0 (IQR: 1.5–5.1) vs 2.6 (IQR: 0.8–10.1) years, respectively (*P* = 0.19), but not different across tumour subtypes.

Metastases were diagnosed on extended radiological imaging that was ordered because of an unusually rapid increase in the size of the APT in 8, increasing hormone levels not explained by the change in pituitary tumour size in 11, as part of scheduled radiological follow-up in 13, and local symptoms/signs or laboratory findings other than increasing hormones (elevated liver enzymes, elevated alkaline phosphatase, decreasing haemoglobin levels) in 16 patients.

The CNS was the first location of metastatic deposits in about half of the patients (Table 5). ACTH-secreting tumours were prone to disseminate to the liver and bone. During longer follow-up, additional metastases were reported in a total of 20 patients, 3 with metastases outside the initially affected region.

**Table 5:** Numbers of tumour subtypes and locations of the first observed metastases in 48 patients with PC.

Tumour subtypes		Brain/cerebellum	Spinal cord	Meninges	Skeletal	Liver	Lung	Lymph nodes
Subtype	*n*							
Corticotroph	22	7	4	3	9^a^	7	1	-
Lactotrotroph	16	8	3	3	2	3	1	4
Non-functioning	7	5	2	1	-	-		1

In some patients, the first detected metastases were present at several locations;

^a^in the bone marrow.

Most metastatic deposits were detected on standard imaging. FDG-PET/CT performed in 23, and ^68^Ga-DOTATATE-PET/CT in 7 patients showed variable uptake into the lesions.

### Treatment

Of the 171 patients, 160 had undergone surgery (median 3; 33 once, 41 twice, and 86 multiple operations). Nine of 11 surgically naive tumours were large prolactinomas or giant invasive NF tumours. A total of 152 patients received RT of whom 55 received 2 or more courses. The first RT was administered as conventional stereotactic (*n* = 99), stereotactic fractionated (*n* = 7), single dose gamma knife (*n* = 28), cyberknife (*n* = 7, of which 4 as hypofractionated up to 5 cycles), LINAC (*n* = 9, given as single doses in 2), and proton beam (*n* = 2). The second (*n* = 55) and third RT (*n* = 10) was given with single dose RT in 34.5% and 50% of patients, respectively.

After the first RT, CR, PR, sSD, and PD occurred in 3.2% (95% CI: 1.3–8.4), 41.9% (95% CI: 33.5–50.9), 47.6% (95% CI: 38.9–56.5), and 7.3% (95% CI: 3.8–13.4), respectively. The response to RT was not related to Ki67 indices. The median time between first and second RT was 5.4 years (IQR: 3.5–8.9 years). The therapeutic effect was similar to the first course.

#### Temozolomide

TMZ was given to 156/171 (91%) of the patients, mostly for 5 days in 28-day cycles, the outcome was reported in 146. Other cytotoxic drugs were used concomitantly in five. The TMZ doses were reduced in 15 and discontinued in 11 because of side effects; low granulocyte count (1), pancytopenia (1), severe depression (1), and not specified in 8. Radiological tumour response was reported as CR in 9.6% patients (95% CI: 5.7–15.6), receiving 12.3 (IQR: 6–13) cycles; PR in 30.1% patients (95% CI: 23.2–38.1), receiving 12 (IQR: 6–18) cycles; SD in 28.1% patients (95% CI: 21.3–36.0), receiving 7 (IQR: 5–16) cycles; and PD in 32.2% patients (95% CI: 25.1–40.3), receiving 5.5 (IQR: 3–9) cycles of TMZ. The response was similar in APT and PC (*P* = 0.94). At least 50% decrease in secreted hormones was achieved in 33.6% of patients with functioning tumours.

The estimated mean duration of the effect of TMZ in responders determined from the date of discontinuation of TMZ to the date of the next treatment (surgery, RT, TMZ re-challenge, other chemotherapy, or peptide receptor radionuclide therapy (PRRT) was 6.4 and 3.3 years after achieving CR and PR, respectively, and 1.4 years in patients with SD.

Combined RT and TMZ was given to 9 patients with clinically functioning tumours, either according to the Stupp protocol (5) or as RT given within 6 weeks prior to cessation of TMZ. Seven patients (77.8%) achieved CR/PR. The tumour Ki67 indices were ≥20% in 5/9 patients and >10% combined with p53 expression or a high mitotic count in 2, and 4% in 1 (no data in 1).

Thirty-one patients received a second TMZ course (Table 6). The median time between first and second course was 2.4 years (IQR: 1.7–4.3) and 4.5 (IQR: 1.3–9.9) years in patients with CR/PR at the first course of TMZ.

**Table 6:** Effect of a second course of temozolomide in 31 patients. No patient achieved complete response (CR) at the second course. Three with PD received TMZ in combination with bevacizumab or chemotherapy.

Effect of first course		Effect of second course		
Response	*n*	PR	SD	PD
CR	4	2	2^a^	0
PR	13	3^b^	3	7
SD	10	1^c^	4^d^	5
PD	4	0	1^e^	4

PD, progressive disease; PR, partial response; SD, stable disease.

^a^SD while on 12 and 24 cycles, respectively; ^b^concomitant with RT in 1; ^c^concomitant with RT; ^d^SD while on TMZ for 3, 12, and 24 cycles, respectively; ^e^while on 10 TMZ cycles.

#### Other treatments

Eleven patients were treated with bevacizumab (Table 7), eight not reported previously. Bevacizumab was given as add-on to TMZ in one case and in combination with 5-FU in another case. Treatment duration ranged from 1 to 28 months. PR, SD, and PD were demonstrated in 1, 3, and 5 patients, respectively. The response was difficult to assess in the remaining two cases.

**Table 7:** Bevacizumab therapy and outcome in 11 patients with APT/PC.

Tumour type	Prior chemotherapy	Combined with	Treatment duration (months)	Outcome	Durability of effect
PC, GH	TMZ (Stupp), TMZ	-	16	Pit. tumour IGF-1, PR;	Sustained at 16 months
				Metastases; SD	Stable 15 months
PC, PRL	TMZ	TMZ (ongoing)	6	SD	7 months
PC, PRL	None	5-FU	28	PD	
APT, ACTH	TMZ	-	9	PD	
PC, PRL,	TMZ	TMZ 2^nd^ trial	9	PD	
PC, PRL	TMZ ×2, CCNU, carboplatin+ paclitaxel	-	5.5	PD	
APT PRL	TMZ	-	1.5	Stopped due to other condition	Not assessed
APT, ACTH	TMZ ×2	-	1	PD	Deceased 3 months after drug stop
^a^APT, silent (6)	TMZ	-	16	SD	Sustained 16 months
^a^PC, silent (7)	TMZ, ICI	-	1.5	SD	Sustained 7.5 months
^a^APT, ACTH (6)	TMZ, TMZ + capicitabine	-	1	PD	

ICI, immune checkpoint blockade; TMZ, temozolomide.

^a^Previously published.

Six patients received immune checkpoint inhibitors (ICI) as second-line therapy (3 not reported previously). One patient achieved PR of the pituitary tumour and the metastatic lesions for 8 months but progressed afterwards (7), and another achieved a transient response (3 months) followed by progression (8). The other four patients had PD, with one case presenting with high mutational tumour burden and positive PD-L1 expression (Table 8).

**Table 8:** Treatment with ICI, tumour characteristics, and outcome in six patients with APT/PC.

Tumour type	MMR mutations	Microsatellite status	Tumour- mutation burden	PD-LI	ICI, dual or PD1 single	Duration, months	Outcome
APT ACTH	No	nd	22.5/Mb	15%	PD1	2	PD
PC ACTH	No (before TMZ)	MS-stable (before TMZ)	Low (before TMZ)	ND	Dual	3	PD
PC, ACTH	MSH6 (after TMZ)	MS-stable	2.5/Mb	ND	Dual	4	PD
^a^APT ACTH (9)	IHC neg. for MSH2, MSH6	nd	Low	neg.	PD1	3	PD
^a^PC ACTH (8)	IHC neg. for MSH6 (after TMZ)	nd	nd	neg.	Dual	14	PD^b^
^a^PC silent PRL (7)	No	nd	6.8/Mb (before TMZ)	<1%	Dual	8	PR for 8 months, then PD

MMR, DNA mismatch repair status; nd, not done; neg, negative; TMZ, temozolomide.

^a^Previously reported; ^b^PD regression of pre-existing liver metastases but appearance of a new liver metastasis, and PD of the pituitary tumour.

PRRT was used in 11 patients (5 not previously reported), predominantly in PRL-secreting and NF tumours (Table 9). Partial regression was achieved in three patients. SD persisting at least 12 months occurred in 1, and another patient had clinically sufficient tumour control for 15 years. Results on prior imaging assessment of somatostatin receptor subtype 2 (SSTR2) were available in six patients, all of which demonstrate tumour uptake of tracer. Of those, three patients achieved partial remission or stabilization.

**Table 9:** Peptide radio-receptor therapy and outcome in 11 patients with APT/PC.

Tumour type	Prior therapy	Assessment SUV max or KS	Type of PRRT	Year: cycles	Outcome
APT silent	TMZ (2×)	^68^Ga-PET: SUV 25	^177^Lu-DOTA-TATE	2020: 4×	PR at 8 months
APT silent	TMZ	Octreoscan: KS ≥ 3	^177^Lu-DOTA-TATE	2016: 4×	PR > 26 months
APT silent	RT	Octreoscan: KS ≥ 3	^177^Lu-DOTA-TOC	2005: 3×; 2015: 2× ; 2020: 1×	SD
APT PRL	TMZ + BVZ	^68^Ga-PET: SUV 8	^177^Lu-DOTA-TATE	2019: 1×	PD
APT PRL	TMZ (2×)	^68^Ga-PET: SUV 6.9	^90^Yttrium-DOTA-TOC; ^177^Lu-DOTA-TATE	2016: 2×; 2016: 1×	PD
APT PRL (10, 11)	TMZ	Octreoscan: KS ≥ 3	^111^In-DTPA-octreotide	2009: 5×	PR at 84 months
APT silent (2)	TMZ	Octreoscan: KS ≥ 3	^90^Yttrium-DOTA-TOC	2013: 2×	SD at 12 months
APT PRL (12)	RT, TMZ	^68^Ga-PET: KS = 3	^177^Lu-DOTA-TATE	2014: 2×	PD
APT PRL (10, 11)	RT	Octreoscan: KS ≥ 3	^177^Lu-DOTA-TOC	2015: 2×	PD
APT TSH (2)	TMZ	Octreoscan: KS ≥ 3	^177^Lu-DOTA-TATE	2012: 2×	PD
PC GH (12)	TMZ	Octreoscan: KS ≥ 3	^90^Yttrium-DOTA-TOC	2008: 1×	PD

APT, aggressive pituitary tumour; BVZ, bevacizumab; KS, Krenning score (grade 2, tumour uptake = normal liver, grade 3, uptake > normal liver, grade 4, uptake > spleen or kidney); mo, months; NG, not given; PC, pituitary carcinoma; PD, progressive disease; PR, partial remission; RT, radiotherapy; SD, stable disease; SUV, standardized uptake values; TMZ, temozolomide.

In another 11 patients, treatment with various cytostatic drugs, the mTOR inhibitor everolimus in 4 patients (3 not reported previously), or tyrosine kinase inhibitors (lapatinib and sunitinib in one patient each, not reported previously) was attempted. No significant responses were reported.

Local treatment of metastases was given in 36 of the 50 PCs. Gross total resections of CNS lesions and/or laminectomies in the spinal cord were performed in 10 and combined with RT in 5 patients. RT was given as local monotherapy in 12 patients with CNS deposits and in 4 with skeletal lesions. Four of 11 patients with liver metastases were treated with laser/radiofrequency ablation/embolization or debulking surgery plus RT. Lymph node metastases were removed in 2/3 patients. The outcome was reported in 33 patients, with CR in 6 and PR in 8 with CNS/spinal cord metastases and 2 with liver metastases. Pain relief was attained in patients with skeletal lesions.

### Causes of death and survival

**Figure 5:**
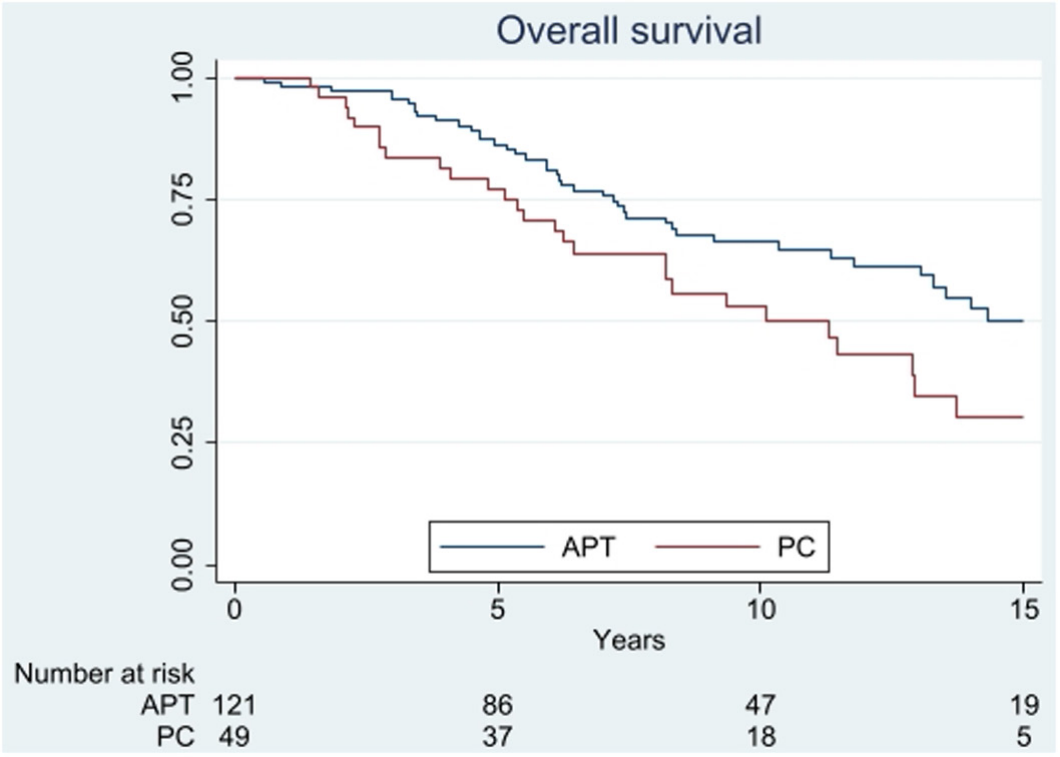
Kaplan–Meier survival curve showing overall survival from diagnosis in a cohort of 170 patients with APT/PC.

**Figure 6:**
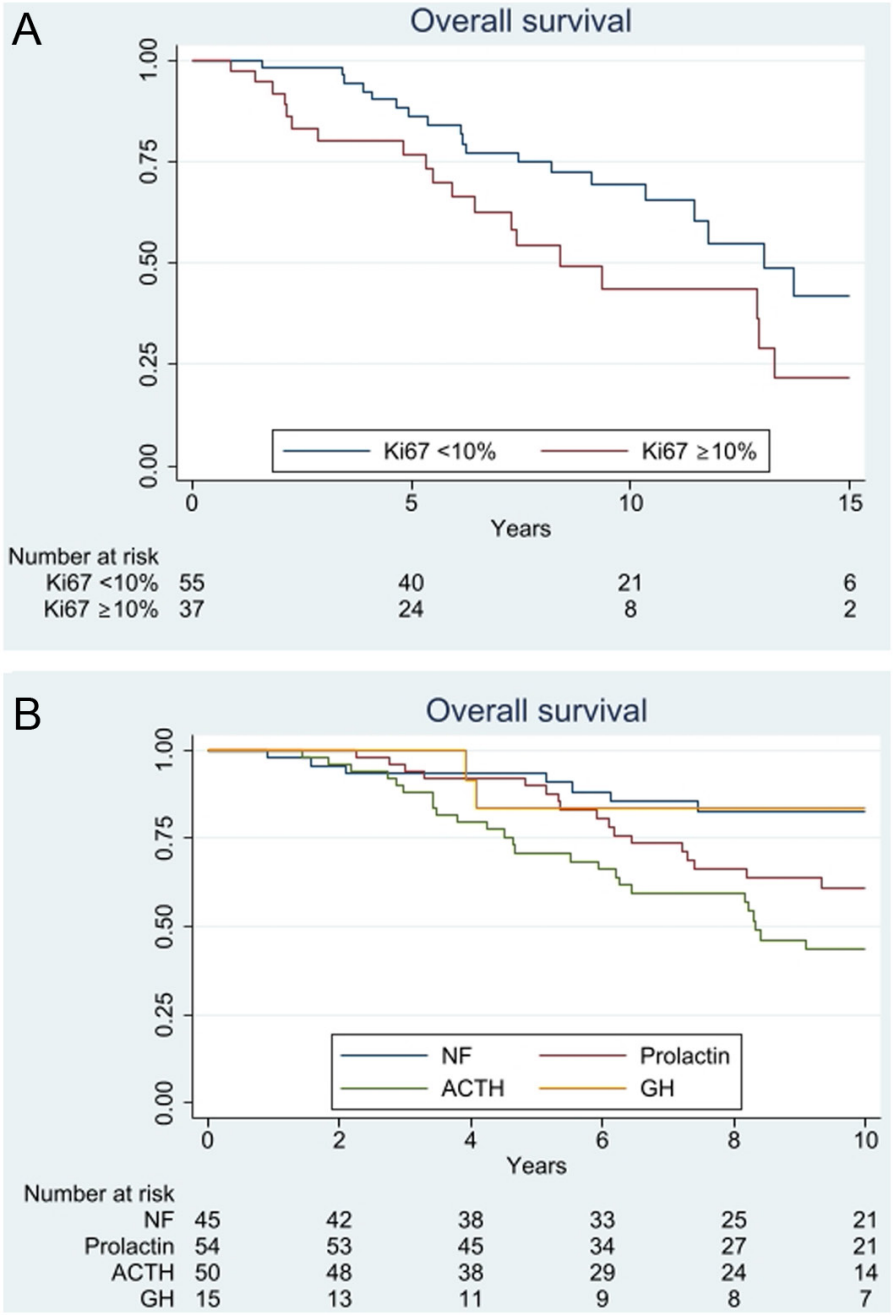
Kaplan–Meier survival curve showing overall survival in APT/PC with respect to tumoural Ki67 indices at the first surgery (A) and hormone secretion at diagnosis (B).

At the study end, 73 patients were deceased, with known causes of death in 69. The majority of deaths (84%) were related to the tumours *per se*, and in another 10% to the treatment; chemotherapy in a patient with severe hypercortisolism, sepsis or haemorrhage after pituitary surgery in 3, gastric ulcer after treatment with prednisolone in 1, bacterial osteomyelitis 3 months after TMZ discontinuation in 1, and unknown cause in 1. The median survival calculated from the time since the first diagnosis of the pituitary tumour was 17.2 and 11.3 years in APT and PC, respectively. The age-adjusted HR was 1.58 (95% CI: 0.98–2.54; *P* = 0.06; Fig. 5). Ki67 ≥ 10% at the first surgery was associated with shorter survival after adjustment for age (HR: 1.53, 95% CI: 0.78–2.99; *P* = 0.21; Fig. 6A). When adjusted for age, ACTH-producing APT/PC were associated with an almost three-fold increased mortality risk compared to NF APT/PC (HR: 2.94, 95% CI: 1.52–5.70; *P* < 0.001). After additional adjustment for Ki67 and APT vs PC, the associated mortality risk was attenuated (HR: 1.54, 95% CI:0.62–3.81; *P* = 0.35; Fig. 6B).

## Discussion

This study describes the largest number of patients with APT/PC ever reported and covers the initial presentation, the evolution over time, and the contemporary management.

### Unsolved challenges in behaviour of APT/PC

More than half of the APT/PC were initially considered clinically benign and later demonstrated aggressive behaviour , in 25% after a decade or more. Although 96% of the tumours were large/giant, 20% were initially not invasive on imaging. In contrast to epidemiological studies showing that the ACTH-secreting adenomas account for about 5% of all pituitary adenomas and 70% are women (13), the APT/PC patients with Cushing’s disease represented 33% and 60% were men. ACTH-secreting tumours in men are less likely to harbour somatic *USP8* mutations that are linked to smaller and less invasive tumours (14, 15). In addition, the transcriptomic analysis revealed enrichment of genes involved in tumour development in corticotroph tumours from men (16). The finding of enrichment for Cushing’s disease is in line with other cohorts of APT/PC (17, 18).

Prolactinomas are the most common secretory pituitary adenomas with a peak incidence in women of childbearing age. After the age of 50 years, there is a change in gender predilection with 88% occurring in men (19). While prolactinomas in younger women are usually small, males typically present with large invasive tumours (19, 20, 21). The properties of the present group of APT/PC prolactinomas are consistent with these observations.

In the surgical series of NF tumours, the gonadotroph subtype is the most common, 58–75% (22), whereas in the present cohort, it accounted for 5–31% (the higher percentage applies assuming that immunostaining for FSH/LH and/or SF-1 was not done in 10 of 14 tumours reported as immunonegative, and if done, would have been positive for FSH/LH and/or SF-1 in all of them). This suggests that silent gonadotroph tumours have a lower risk of becoming aggressive compared to silent tumours of other lineages.

Five out of 13 silent corticotroph tumours (SCA) became ACTH-secreting. The shift preceded or occurred concomitantly with a change from a benign to clinically aggressive course suggesting that these events were linked. In comparison, such a shift is less common in non-aggressive SCA, reported in 3/75 (23), 3/44 (24) and in 1/39 (25), respectively. In the study by Righi and co-workers (24), the three SCAs changing into Cushing’s disease had acquired higher immune expression and mRNA levels of prohormone convertase 1/3, an enzyme which processes pro-opiomelanocortin to ACTH. Furthermore, amongst six initially silent somatotroph APT/PC, two (33%) later evolved into GH secreting*,* a shift rarely reported in benign silent somatotrophs (26). Thus, a change in hormone secretion should alert the clinician to a potentially aggressive course.

Assessment of the tumour proliferative potential by mitotic count and Ki67 index is recommended for consideration of clinically aggressive tumours (1). About 40% of APT/PC in this survey had Ki67 levels ≥ 10%, to be compared with 3% of 374 tumours in the Lyon surgical series (27). A proportion of both APT and PC had extensive p53 immunostaining, another potential indicator of aggressiveness, which underlines the similarity between these tumours (27, 28).

*TP53* is a tumour suppressor and one of the most frequently mutated genes in cancer. Mutations are accompanied by high nuclear expression of the encoded p53 protein, due to decreased protein degradation, but may also result in complete absence of the protein. In 266 pituitary tumours reported in the German Pituitary Register (29), p53 staining in more than 10% of the tumour cells was observed only in a subgroup of 78 considered to be atypical tumours based on the World Health Organization 2004 classification (30). In neuroendocrine gastroenteropancreatic carcinomas, more than 10% of positive cells reflect *TP53* mutations (31). In pituitary tumours, consensus on a cut-off level has not been established due to technical problems with the p53 immunohistochemistry. *TP53* mutations, previously considered rare in APT/PC, were recently reported in 20–30% of *USP8* WT corticotroph macroadenoma cohorts (32, 33) characterized by recurrence after surgery and in APT/PCs (34). The number of APT/PC with *TP53* mutations could be underestimated and is a subject of future research.

BADX results in the expansion of ‘ordinary’ pituitary corticotroph tumours in 28–53% of patients (4). Aggressive corticotroph tumours have been suggested to be particularly prone to progress after BADX (4). However, neither the presence of mitosis nor a high Ki67 index was found to predict tumour progression (35). Compared to the ‘ordinary’ ACTH-secreting microadenomas, ACTH-secreting macroadenomas have attenuated ACTH responses to CRH (36) and show less glucocorticoid suppressibility (36, 37). Possibly, the pituitary-glucocorticoid feedback is equally attenuated in some aggressive ACTH-secreting macro/giant tumours, making them less susceptible to a potential influence of BADX. Based on the participants’ judgements, in combination with the reported changes in ACTH levels after BADX, accelerated pituitary tumour growth may not be the rule.

In the 50 patients with PCs, metastases have detected a median of 6.3 years after the initial diagnosis of the pituitary tumour and a median of 3.8 years after the recognition of the tumour as being aggressive. Whereas the CNS was affected in all tumour subtypes, the ACTH-secreting tumours were particularly prone to disseminate systemically, mostly to bone and liver. The finding that merely a third of the metastases were symptomatic suggests that carcinomas may be more common than previously thought with silent deposits being unrecognized.

### Effectiveness of current and emerging treatment strategies in stabilizing the disease

Similar to the first ESE survey (2), an objective response to TMZ monotherapy was described in 40% of patients and few patients received TMZ combined with other chemotherapy. TMZ given concurrently with RT, e.g. the ‘Stupp protocol’, is the standard treatment of glioblastomas based on the radio-sensitizing effect of TMZ *in vitro* and in experimental animals (38). Limited experience of this protocol in APT/PC has indicated a better effect compared to TMZ alone (3). In the present survey, this combination was primarily given to patients with highly proliferative tumours. The use of the Stupp protocol in APT/PC could be advantageous in selected patients but should be further evaluated in larger cohorts.

Previous analyses suggested that a second TMZ course often fails (3). In this survey, retained sensitivity to a TMZ rechallenge (CR/PR) occurred in 5/17 tumours that responded to the first treatment period and in 1/11 with SD. In the latter group, 4/11 attained a further period of disease stabilization for at least 1 year. Given the few alternative treatment options, a second TMZ course could be considered in patients who respond well to the first one.

Bevacizumab is a humanized anti-VEGF antibody that inhibits tumour microvessel formation. Previously, 12 patients with APT/PC treated with the drug have been reported (6, 7, 8, 39, 40, 41, 42, 43, 44, 45, 46), in 3 cases combined with RT and TMZ (42, 43, 44). When given as monotherapy (8 evaluable patients), mostly as second-line chemotherapy, disease stabilization was achieved in 4; in 1 PC, the effect was ongoing during a treatment period of 26 months (37), in another 3 for at least 6 months (6, 7, 39). Two of three patients given bevacizumab combined with TMZ and RT achieved objective radiological responses (42, 44), whilst SD for many years occurred in the third (40). The present survey contributes another four patients in whom a drug effect *per se* was possible to evaluate (PR 1, PD 3). Taken all treated patients together, radiological response was achieved in 1 and SD for at least 6 months in 4 of 12 patients treated with bevacizumab monotherapy. This outcome suggests that the drug might be considered as second-line therapy.

The effect of ICI relies on complex crosstalk between tumours and immune-competent cells in the tumour microenvironment. Treatment has been tried only in few patients with APT/PC (7, 8, 9, 47, 48, 49, 50, 51), with the 3 new reported herein, all non-responders, the total number amounts to 15. Clinically meaningful responses (CR, PR, or SD of longer duration), in the absence of severe toxic effects, has been achieved in 7/15. Rapid progression, with tripling of tumour volume over 3 months, was reported in 1 case (9). An accelerated tumour growth rate (hyperprogression) seems more common during treatment with ICI compared to other chemotherapy (52). Two ongoing clinical trials (NCT02834013, NCT04042753) will further elucidate the role of ICI in the treatment algorithm.

PRRT is a molecularly targeted radiation therapy. The radiolabelled peptide binds to SSTR2 on tumours. In this survey, PRRT was given to NF tumours and Pit-1-positive tumours. All demonstrated significant SSTR2 binding, but radiological response (at best PR) was demonstrated in 3/10 evaluable patients. This illustrates that properties other than SSTR2 binding are needed for tumoricidal effects. Including other published cases (53, 54, 55, 56), PR has been attained in 4/18, and disease stabilization for at least a year in another 4 treated patients. However, four of the eight did not have prior chemotherapy potentially reflecting a selection bias of less severe tumours.

Loco-regional treatment of metastases generally offered pain relief which is an important treatment goal. Since the metastatic burden was highly variable, an effect of surgery vs RT on survival was not possible to evaluate.

In a case series of 15 PCs reported in 1997, prior to the era of TMZ, 66% were deceased after 1 year and 80% after 5 years (17). In a recent review of PC reported between 1990 and 2016, information on survival was available in 58 patients (57). Of the 32 who were deceased at the time of publication, the average time to death was 10 months, but only a third of the deceased patients had been treated with TMZ. The improved survival of patients with PC in our cohort compared to early publications likely reflects advancements in management.

There are several limitations with the present survey which is based on retrospective data collection. The patients were managed in many centres, the histopathological techniques were not standardized, and the results were not established by two independent pathologists in a blind manner. Tumour responses were not centrally evaluated.

In conclusion, APT/PCs display a spectrum of unusual properties which distinguish them from pituitary adenomas and demonstrate a wide range of behaviour with respect to disease progression. TMZ remains the first chemotherapy of choice, with consideration of the administration of concurrent RT in selected patients. TMZ re-challenge could be tried in patients with an objective response to the first course. Bevacizumab and ICI have both resulted in clinically meaningful and durable responses in small proportions of patients and represent options for progression after TMZ. The place of PRRT remains to be established and the potential use at an earlier stage needs further investigation. Patients with APT/PC need to be managed within expert multidisciplinary teams. Hopefully, increased access to tumour molecular data will assist clinicians in individualizing treatment approaches and improving patient outcomes.

## Supplementary Material

Supplemental Table. Characteristics of 87 patients (cohort 1) from the ESE survey 2016 and 83 patients (cohort 2) included in the ESE survey 2020-2021

## Declaration of interest

This study was supported by the European Society of Endocrinology. G Raverot and O M Dekkers are on the editorial board of the *European Journal of Endocrinology.* G Raverot and O M Dekkers were not involved in the review or editorial process for this paper, on which they are listed as authors.

## Funding

The study was supported by a research grant from Ipsen, Rare Diseases/Endocrinology to ESE.
