# Supplementary material for: Aggressive pituitary tumours and carcinomas, characteristics and management of 171 patients
Source: Eur J Endocrinol. 2022 Aug 26;187(4):593–605. doi: 10.1530/EJE-22-0440 (PMC9513638; doi:10.1530/EJE-22-0440)
Supplement: Supplemental Table. Characteristics of 87 patients (cohort 1) from the ESE survey 2016 and 83 patients (cohort 2) included in the ESE survey 2020-2021 [file supplementary_table_1.pdf]

Supplemental Table. Characteristics of 87 patients (cohort 1) from the ESE survey 2016 and 83 patients (cohort 2) included in the ESE survey 2020-2021

	No	Yes	Missing	p-value
	N=83	N=87	N=1	
APT or PitCarc				0.70
APT	57 (69%)	63 (72%)	1 (100%)	
PitCarc	26 (31%)	24 (28%)	0 (0%)	
AgeAtDx	48.5 (16.1)	43.1 (15.0)	47.0 (.)	0.079
Patient sex				0.69
Female	33 (40%)	30 (34%)	0 (0%)	
Male	49 (59%)	57 (66%)	1 (100%)	
Missing	1 (1%)	0 (0%)	0 (0%)	
Initial Hormone production				0.79
None	20 (24%)	24 (28%)	1 (100%)	
Prolactin	26 (31%)	28 (32%)	0 (0%)	
ACTH	24 (29%)	27 (31%)	0 (0%)	
FSH	0 (0%)	1 (1%)	0 (0%)	
GH	11 (13%)	4 (5%)	0 (0%)	
TSH	1 (1%)	2 (2%)	0 (0%)	
Unknown	1 (1%)	1 (1%)	0 (0%)	
MRatDx				0.86
Microadenoma	2 (2%)	4 (5%)	0 (0%)	
Macroadenoma	58 (70%)	65 (75%)	1 (100%)	
Giant adenoma (largest diameter ≥40 mm)	20 (24%)	17 (20%)	0 (0%)	
Missing	3 (4%)	1 (1%)	0 (0%)	
TumorPartHereditarySY				0.37
No	81 (98%)	82 (94%)	1 (100%)	
Yes	2 (2%)	1 (1%)	0 (0%)	
Unknown	0 (0%)	4 (5%)	0 (0%)	
Invasive at diagnosis Y_N				0.88
Not invasive	13 (16%)	13 (15%)	0 (0%)	
Invasive	50 (60%)	57 (66%)	1 (100%)	
Missing	20 (24%)	17 (20%)	0 (0%)	
Was Pituitary Radiotherapy performed?				0.81
No	10 (12%)	8 (9%)	0 (0%)	
Yes	72 (87%)	79 (91%)	1 (100%)	
Missing	1 (1%)	0 (0%)	0 (0%)	
Was pituitary surgery performed?				0.94
No	5 (6%)	6 (7%)	0 (0%)	
Yes	78 (94%)	81 (93%)	1 (100%)	
